# Analysis of Surface Plasmon Resonance Curves with a Novel Sigmoid-Asymmetric Fitting Algorithm

**DOI:** 10.3390/s151025385

**Published:** 2015-09-30

**Authors:** Daeho Jang, Geunhyoung Chae, Sehyun Shin

**Affiliations:** 1 School of Mechanical Engineering, Korea University, Seoul 136-701, Korea; E-Mail: jangdh@korea.ac.kr; 2 College of Information and Communication Engineering, Sungkyunkwan University, Suwon 440-476, Korea; E-Mail: chkh86@skku.edu

**Keywords:** surface plasmon resonance, fitting algorithm, sigmoid-asymmetric, critical angle, resonance angle

## Abstract

The present study introduces a novel curve-fitting algorithm for surface plasmon resonance (SPR) curves using a self-constructed, wedge-shaped beam type angular interrogation SPR spectroscopy technique. Previous fitting approaches such as asymmetric and polynomial equations are still unsatisfactory for analyzing full SPR curves and their use is limited to determining the resonance angle. In the present study, we developed a sigmoid-asymmetric equation that provides excellent curve-fitting for the whole SPR curve over a range of incident angles, including regions of the critical angle and resonance angle. Regardless of the bulk fluid type (*i.e.*, water and air), the present sigmoid-asymmetric fitting exhibited nearly perfect matching with a full SPR curve, whereas the asymmetric and polynomial curve fitting methods did not. Because the present curve-fitting sigmoid-asymmetric equation can determine the critical angle as well as the resonance angle, the undesired effect caused by the bulk fluid refractive index was excluded by subtracting the critical angle from the resonance angle in real time. In conclusion, the proposed sigmoid-asymmetric curve-fitting algorithm for SPR curves is widely applicable to various SPR measurements, while excluding the effect of bulk fluids on the sensing layer.

## 1. Introduction

Since the first observation using surface plasmon resonance (SPR) sensors by Wood in 1902 [1,2], SPR sensors have emerged as popular analysis tools for bio-molecules, used label-free to detect changes in the refractive index or thickness of an adsorbed layer on or near the sensing film of the SPR sensor with a high sensitivity in real time [3,4,5,6,7,8,9]. However, the performance of the SPR measurement still requires improvement for reliable and high-speed data analysis. In fact, the curve-fitting of the SPR curve is an important and unique process to determine the performance of the SPR sensing, distinguishing the SPR measurement from other direct measurements using cantilever, fluorescence, and electrochemical sensors.

For a typical angular interrogating SPR system, a SPR curve indicating the reflectance intensity *versus* the incident light angle provides a fundamental concept to analyze the binding kinetics of analytes on a sensor film according to changes in the refractive index [10]. SPR sensors generally monitor the changes of reflectance intensity over a range of incident angles when target-molecules interact on the sensing surface. The angle yielding the minimum light intensity on an SPR curve is denoted as the resonance angle, which is carefully determined with curve fitting for an SPR curve in a small range of incident angles. For the accurate measurement of the resonance angle from an SPR curve, several fitting methods have been proposed, such as the polynomial fits [11,12], centroid method [6] and parabolic fit [13,14]. Additionally, optimal linear method [15], asymmetric method [10] and signal processing methods of the SPR signals [16] were proposed. Also to determine the SPR line in the SPR image, researches utilizing Radon transform were introduced [17,18,19,20,21]. In particular, the asymmetric fitting method determines the resonance angle very accurately using a simple equation derived from the complicated multi-layer Fresnel equation.

**Figure 1:**
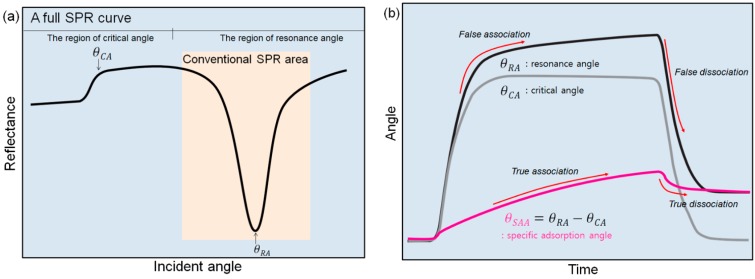
Concept schemes of the proposed sigmoid-asymmetric fitting method: (**a**) A full SPR curve including both regions with critical angle and resonance angle. (**b**) Sensor grams indicating the changes in resonance angle, critical angle and specific adsorption angle, respectively. Our novel method can monitor the change in the specific adsorption angle ($\theta_{SAA}$) by simultaneously monitoring the change in the resonance angle ($\theta_{RA}$) and the change in the critical angle ($\theta_{CA}$).

However, conventional curve-fitting methods have been used for determining the change in the resonance angle in short ranges of the incident angle with wedge-shaped beam type angular interrogation SPR spectroscopy, which is the most popular and appropriate SPR system for real-time monitoring, as shown in Figure 1a. When the targeted molecular interaction is measured by SPR spectroscopy, the real-time results obtained by the change of the resonance angle are also affected by the bulk fluid, which causes a bulk sensor refractive index. In fact, the existence of bulk fluid molecules around a sensing range cannot be avoided and should be excluded in the measured results. Without considering the undesired the effect of bulk fluid molecules, it is difficult to accurately evaluate the net binding kinetics of target molecules by analyzing only the resonance angle. 

Conventional SPR devices have adopted a reference channel to remove noise signals caused by the bulky effect of the flowing medium. In order to add a reference channel in a SPR sensor design, it is necessary to give up a main sensing channel on a limited sensor area. Furthermore, noise signals vary greatly with referencing approach [22]. However, these noise signals can be effectively removed by obtaining the critical angle and resonance angle simultaneously without a reference channel when non-specific binding is absent. It is known that the critical angle is related to the refractive index of the surrounding medium [23]. Thus, if the medium is changed, the critical angle would be shifted and the resonance angle also would be shifted, correspondingly. Therefore, the capability to determine both the resonance and critical angles from a SPR curve over an entire range of incident angles is highly required. A successful curve-fitting method for a whole SPR curve can provide both critical and resonance angles. Then, the change of angle on specific adsorption of the analyte can be achieved by subtracting the critical angle from resonance angle in real-time as shown Figure 1b. However, an SPR curve in an entire range of incident angles cannot be easily fitted because of the complicated shape of the curve. The conventional fitting methods are not suitable for fitting the entire SPR curve over a range of incident angles. Although a multi-layer Fresnel equation with curve fitting [24] can determine the critical angle and resonance angle accurately, this has rarely been practically applied because the properties are not fully available and it requires a long computation time [10].

In this study, we proposed a novel fitting algorithm based on a sigmoid-asymmetric equation that can fit an SPR curve over an entire range of incident angles. The proposed curve-fitting equation is a formulation combining the sigmoid-equation and asymmetric equation. The former equation determines the critical angle, whereas the latter determines resonance angle. Using this curve-fitting method, one can rapidly determine a critical angle as well as a resonance angle. The present analytical results were compared with those for the asymmetric and polynomial equation based fitting methods. In the present study, we also confirmed the feasibility for evaluating of specific adsorption of an analyte on a sensor chip by monitoring in real time the specific adsorption angle subtracting the critical angle from resonance angle with correlation constants.

## 2. Experimental

### 2.1. Instrumentation

**Figure 2:**
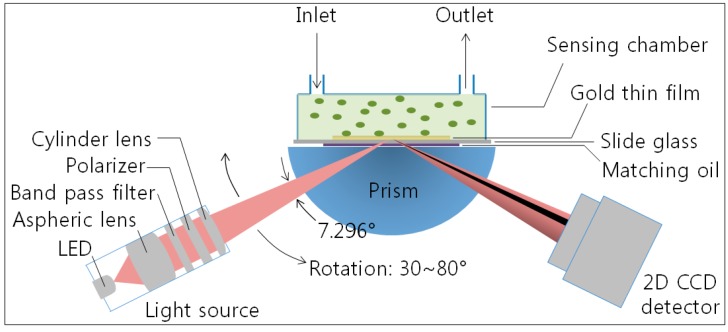
Schematic of our homemade SPR system based on angular interrogation of Kretchmann configuration. The p-polarized wedge-type incidence beam with a range of the incident angle of 7.296° passes through a band-pass interference filter (770 ± 10 nm) and is directed to the SPR sensor chip through a cylindrical prism (BK7, *n* = 1.5125 at 770 nm). Then, the intensity of the reflected light beam is monitored using a two-dimensional complementary metal oxide semiconductor (2D-CMOS) image sensor (IDS Co.) with a 1.41 cm sensing area (1280 × 1024 pixels).

We fabricated a lab-made wedge-shaped type angular interrogation-based SPR spectroscopy for signal detection. The equipment includes a light source, prism, detector and signal analysis software, liquid handling system with a peristaltic pump and a degasser and the flow cell. A schematic of our angular-interrogation-based Kretchmman-configuration SPR system is presented in Figure 2. A slide glass with a sputtered gold layer (50 nm of Au on 2 nm of Cr) on one side together with the flow cell is pressed against the prism coated with an index matching fluid in order to ensure continuous proceeding of the light. We used a 770 nm light-emitting diode (Opnext Inc., Tokyo, Japan) as a light beam in our system. The *p*-polarized wedge-type incidence beam with a range of the incident angle of 7.296° (1 pixel = 0.0057°) passes through a band-pass interference filter (770 ± 10 nm) and is entered to the SPR sensor chip through a half-cylindrical prism. Then, the intensity of the reflected light beam is monitored using a two-dimensional complementary metal oxide semiconductor (2D-CMOS) image sensor (IDS Co., Obersulm, Germany), which has a 1.41 cm sensing area (1280 × 1024 pixels). The image sensor is located immediately in front of the prism, and it allows the SPR system to be fabricated without any other lenses. Our system also has a rotation stage, which can control the incident angle from 35° to 85° as need for the various samples, including gas and liquid solutions. The flow cell is composed of independent three channels with dimensions of 5 mm (l) × 1 mm (w) × 0.2 mm (h) and fabricated from polyether ether ketone (PEEK) plastic. PEEK is used because it has excellent mechanical and chemical resistance properties. The sample solution is driven by peristaltic pump into flow cell and passes through a degasser in order to remove bubbles in the solution before entering the flow cell.

### 2.2. Image Processing

**Figure 3:**
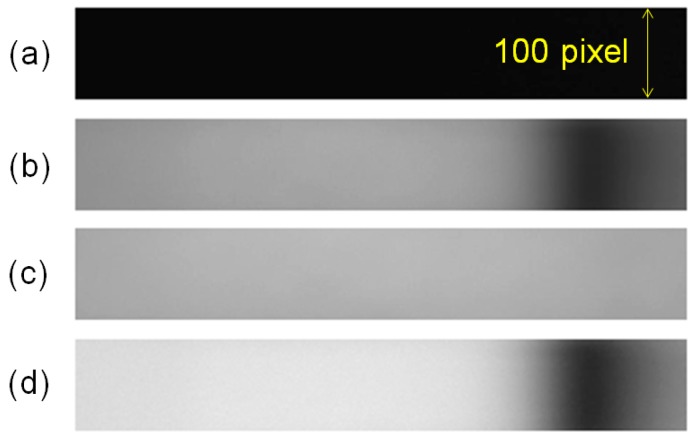
Images from a 2D CMOS image sensor: (**a**) The dark image is obtained when the incident light is turned off. (**b**) The TE-mode. (**c**) TM-mode images are obtained from the light-on mode when the polarizer is in TE-mode and TM-mode after the running buffer is injected on a gold sensor chip. (**d**) The final image is obtained after image processing using (a), (b), and (c).

A final image for the curve fitting is acquired from three images—a dark image, TE-mode image, and TM-mode image—using a self-made MATLAB-based program. The dark image is obtained when the incident light is turned off, while the TE-mode and TM-mode images are obtained from the light-on mode when the polarizer is in the TE-mode and TM-mode, after the running buffer is injected on a gold sensor chip. We processed these three images using the following methods. Firstly, the intensity of the dark image is subtracted from the TE-mode and TM-mode images in order to remove the noise signal in the dark condition. Then, the final image is derived by dividing the subtracted TM-mode image by the subtracted TE-mode image. Figure 3 shows four images obtained from a 2D CMOS image sensor.

$$\text{Final image }=\;\frac{TM\;mode\;image-Dark\;image}{TE\;mode\;image-Dark\;image}$$

### 2.3. Fitting Algorithm Based Sigmoid-Asymmetric Equation

**Figure 4:**
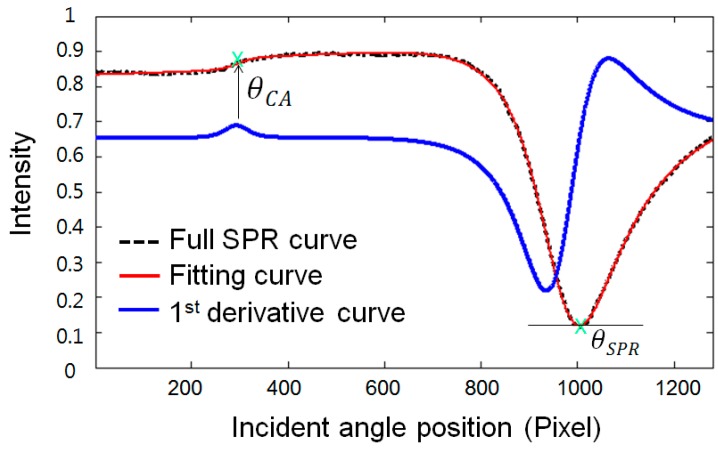
Determination of a resonance angle and a critical angle using sigmoid-asymmetric curve fitting: full SPR curve (black dotted line) plotted from the final image is first fitted by the sigmoid-asymmetric equation (red solid curve), and the resonance angle and critical angle are simultaneously and automatically determined after calculating the 1st derivative (blue solid line).

Figure 4 shows the proposed concept of the sigmoid-asymmetric curve-fitting algorithm. A full SPR curve denoted by the black dotted line in Figure 4 is acquired by plotting the average intensity values of 100 rows for each column in the final image, which is processed using the method described in Section 2.2. Then, the full SPR curve is fitted by the proposed sigmoid-asymmetric equation of Equation (1):

(1) $$R\left(X\right)=\left(\frac{A\times \left[1-\left\{B+C\times \left(X-D\right)\right\}\right]}{{\left(X-D\right)}^{2}+E^{2}}\right)+\left(\frac{F}{1+e^{G\times \left(X-H\right)}}\right)+\left(I\times X\right)$$

This equation is a formula combining the asymmetric function Equation (2) [10] with an equation modified from the sigmoid function Equation (3) [25].

The asymmetric function contributes to the determination of the optimal resonance angle on the right side of the full SPR curve, and the sigmoid function allows the determination of the optimal critical angle on the left side:

(2) $$R\left(X\right)=\left(\frac{A\times \left[1-\left\{B+C\times \left(X-D\right)\right\}\right]}{{\left(X-D\right)}^{2}+E^{2}}\right)$$

(3) $$f\left(x\right)=\left(\frac{1}{1+e^{-x}}\right)$$

In Equation (1), the parameters A, B, C, D, and E are real and constant values needed to fit the right side of the full SPR curve to the asymmetric function, and the parameter X represents the incident angle [11]. The parameters F, G, and H are real and constant values needed to fit the left side of the full SPR curve to the modified sigmoid function, and the parameter I is a real and constant value representing the tilt of the modified sigmoid function. The red solid line of Figure 4 is the fitting curve obtained using the proposed sigmoid-asymmetric equation. The proposed method determines a resonance angle, which is a response angle position to the minimum reflectance on the fitting curve obtained by the sigmoid-asymmetric equation. Moreover, it simultaneously determines the critical angle that is a response angle position to the maximum value of the 1st derivative curve in the region of the critical angle, as indicated by the blue solid line of Figure 4.

### 2.4. Sample Preparation and Measurements

*Chemicals*: Glycerol, bovine serum albumin (BSA), phosphate-buffered saline (PBS) were purchased from Sigma, Inc. (St. Louis, MO, USA).

*Gold sensor chip*: The glass slide (20 mm × 10 mm × 0.55 mm) was from Asahi Glass, Inc. (Tokyo, Japan). The chrome and gold sputtered on the slide with 2 nm and 48 nm of thickness.

*Glycerol solutions*: Distilled ionized water (DIW) and glycerin solutions of 1%, 2%, 3%, 4% and 5% in DIW were prepared and measured with our SPR instrument to know the relationship between critical angle and resonance angle. First, the DIW was injected into a microchannel on the gold sensor chip for a baseline with flow rate of 40 μL/min. Subsequently, the glycerol-water mixture solutions were loaded at 500 s intervals.

*BSA adsorption*: BSA of 5 μg/mL in a 1 × PBS with 1.5% glycerin was prepared for protein adsorption in real time to confirm the feasibility of removing the bulk fluid effect. Here, the diluted BSA and glycerin were used as a model protein for adsorption on the gold sensor chip and for artificially changing the bulky refractive index around the sensor film. First, the 1 × PBS was injected into a micro channel on the gold sensor chip for a baseline with flow rate of 40 μL/min. Then, the BSA solution was loaded and then rinsed by 1 × PBS with same flow rate.

## 3. Results and Discussion

**Figure 5:**
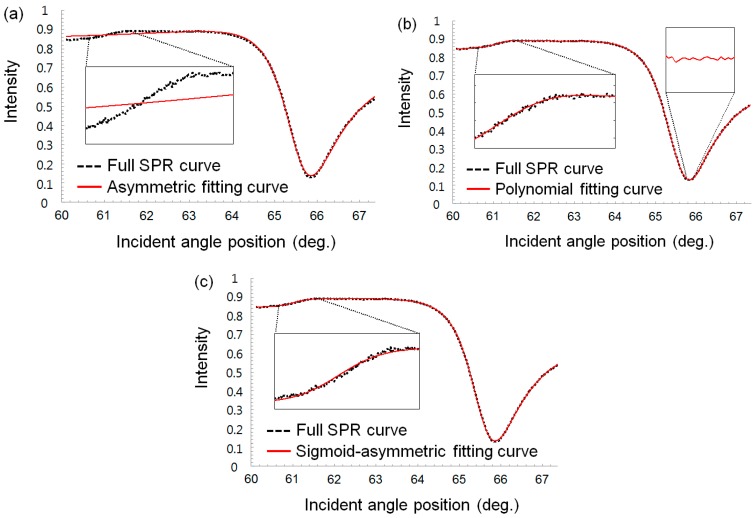
SPR curve-fitting results measured in water (black dot line: SPR curve, red solid line: fitting curve): (**a**) Asymmetric fitted SPR curve. (**b**) 24th-order polynomial regression-fitted SPR curve. (**c**) Sigmoid-asymmetric fitted SPR curve.

**Figure 6:**
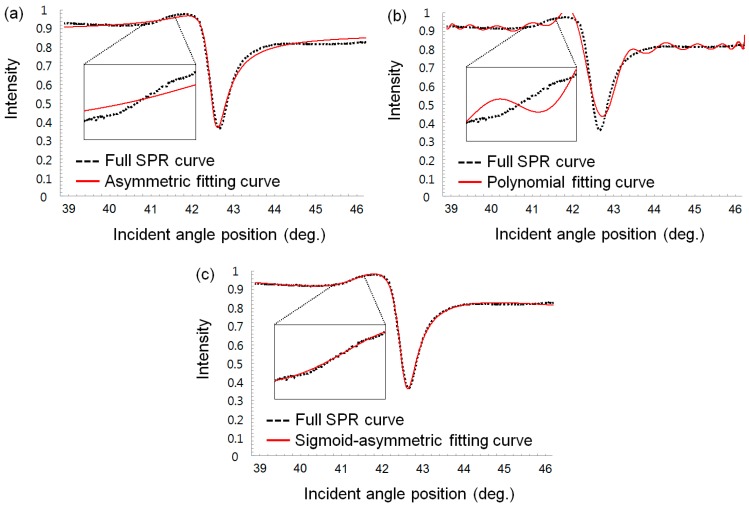
SPR curve-fitting results measured in air (black dot line: SPR curve, red solid line: fitting curve): (**a**) Asymmetric fitted SPR curve. (**b**) 24th-order polynomial regression-fitted SPR curve. (**c**) Sigmoid-asymmetric fitted SPR curve.

Using a MATLAB-based program developed in-house, we compared the curve fitting results for a SPR curve using three different methods: the asymmetric, 24th-order polynomial regression and sigmoid-asymmetric equations. We excluded the centroid and 2nd order polynomial method, which are also popular methods used in SPR spectroscopy, from our comparison experiments because those are local curve fitting methods with threshold values on SPR curves. In Figure 5 and Figure 6, a full SPR curve for the fitting was obtained by a wedge type angular interrogating SPR sensor system with air and water as bulk fluids on a gold sensor chip, and each fitting curve was plotted, respectively. 

Herein, air and water were used to confirm the feasibility in both gas and liquid phases, and with dry and wet samples. Also both fluids are good readily available examples to compare the fit quality according to the curve shapes because the shape of each SPR curve in both bulk fluids is very different. The performance of curve-fitting results with both bulk fluids was compared in the critical angle region and resonance angle region, respectively. The two regions that depict each angle were divided at a criterion angle, which was carefully determined. The criterion angles for water and air were 600 and 550 pixel of the total incident angle, respectively. Available fit quality parameters, including the coefficient of determination (CD), error variance (EV), and angle positions determined by each fitting method, are summarized in Table 1. The coefficient of determination, *R*^2^, indicates how well the experimental data fit the equation models. The EV is used to be defined as follows:

(4) $$\frac{1}{N}\overset{N}{\underset{i=1}{\sum }}{\left(E_{i}-\bar{E}\right)}^{2}$$

where *E_i_* = *X_i_* − *Y_i_* and $\bar{E}$ is average of *E_i_* at each incident angle position. Here, the *X_i_* is the intensity of the fitting curve and *Y_i_* is that of an experimentally obtained SPR curve at each incident angle position *i*. The resonance angle position was determined at the position where the minimum intensity yields a resonance angle region, whereas the critical angle position was determined at the position yielding the maximum of the 1st derivative SPR curve in the critical angle region.

In Figure 5a and Figure 6a, the red solid lines represent the fitting curves obtained using the asymmetric equation. In the region of the resonance angle (pixel number > 600), the SPR curve was well fitted with the fitting curve. The CDs for water and air was 0.999 and 0.981, respectively and these values are fairly good results compared with sigmoid-asymmetric results (0.999 and 0.997, respectively), as listed in Table 1. However, the region of the critical angle on the left side was not fitted well, as shown Figure 5a and Figure 6a. Consequently, the asymmetric curve-fitting method could not determine the critical angle position except for resonance angle. Thus, the resonance angles determined using asymmetric curve-fitting method were 66.6303° and 42.5929° for water and air, respectively, which agreed well with the sigmoid-asymmetric results (66.6189° and 42.6210°, respectively). 

**Table 1:** Calculated statistical results including error variance and coefficient of determination and both angle positions obtained by fitting methods based on asymmetric, 24th-order polynomial and sigmoid-asymmetric equation for a full SPR curve. The SPR curve was experimentally obtained with water and air as bulk fluids on the sensing film, where *N.A*.: not available.

Bulk Fluid	Region	Fitting Method	Coefficient of Determination	Error Variance (×10^−4^)	Angle (°)
Water	Resonance angle	Asymmetric	0.999	0.268	66.6303
		24th order polynomial	0.999	0.020	66.6303
		Sigmoid-asymmetric	0.999	0.037	66.6189
	Critical angle	Asymmetric	0.536	1.189	*N.A*
		24th order polynomial	0.998	0.004	*N.A*
		Sigmoid-asymmetric	0.995	0.014	61.8309
Air	Resonance angle	Asymmetric	0.981	3.177	42.5929
		24th order polynomial	0.947	8.881	42.6955
		Sigmoid-asymmetric	0.997	0.499	42.6100
	Critical angle	Asymmetric	0.773	0.960	*N.A*
		24th order polynomial	0.412	2.488	*N.A*
		Sigmoid-asymmetric	0.985	0.065	41.3208

In Figure 5b and Figure 6b, the fitting curves obtained using the 24th-order polynomial regression equation exhibited a very different appearance depending on both bulk fluids. First, the fitting curve for the water condition on the gold sensor chip agreed well with the full SPR curve, but that for air was fitted poorly. The statistical results in Table 1 also clearly indicate the poor curve-fitting for air compared with water. Secondly, even for water, close inspection of the resonance angle region reveals that the fitted SPR curve is not smooth due to the characteristics of the polynomial equation. This unsmooth fitted curve makes determining the minimum resonance angle difficult and subsequently degrades the reproducibility regarding the determination of the resonance angle. 

In contrast, the fitting curves obtained using the proposed sigmoid-asymmetric equation almost perfectly matched the whole SPR curve over a range of incident angle, as indicated by Figure 5c and Figure 6c. Immediately after curve-fitting with a sigmoid-asymmetric equation, both the critical and resonance angles could be determined. The determined resonance and critical angles were 66.6189°, 61.8309° and 42.6100°, 41.3208° with water and air, respectively. The determined critical angles almost coincide with theoretical critical angles (61.6265° and 41.3049° with water and air). The quality of the determined angle positions can be verified by the statistical results of curve-fitting in Table 1. The CDs were nearly 1 and the EVs were also relatively small compared with others, regardless of the bulk fluid types.

As summarized in Table 1, the three fitting methods agreed fairly well in determining resonance angle positions with water as a bulk fluid. The CDs for the three methods are greater than 0.999 and the angle is in the range of 66.6189°–66.6303°. However, for air, the quality of the curve-fitting was generally degraded for all three methods. In particular, the CD and EV for the polynomial method are significantly degraded, and subsequently the corresponding resonance angle (42.6955°) was different from that of other methods (42.5929°, 42.6100°). Meanwhile, the critical angle cannot be determined by any curve-fitting methods except the sigmoid-asymmetric methods, as listed in Table 1. The asymmetric method was unsuitable for determining the critical angle, yielding poor values of the CD and EV for both water and air. Also, the polynomial method was not able to determine the critical angle even though the values of the CD and EV are fairly good for water as a bulk fluid. It is worthy to note that the sigmoid-asymmetric method yielded fairly good fitting results for both water and air. Therefore, the sigmoid-asymmetric method was the only one to fit a whole SPR curve with high quality and thus determine both the resonance and critical angles with precision.

**Figure 7:**
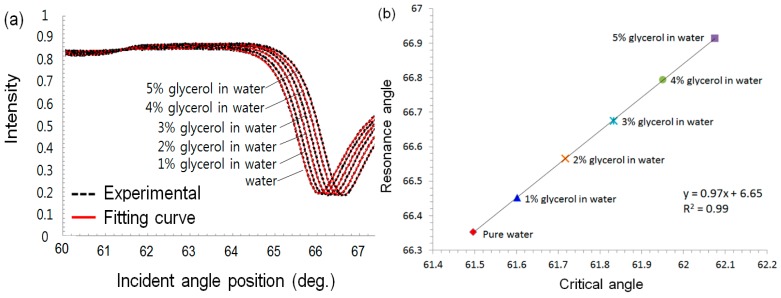
(**a**) Full SPR curves (black dotted lines) measured for a DIW and a glycerol-water solution with a concentration in the range of 1% to 5% as a refractive-index solution and the curves (red solid line) fitted by sigmoid-asymmetric method. (**b**) Correlation graph between the changes in the critical angle and resonance angle caused by the change in the bulky refractive index due to glycerol-water solutions.

In order to monitor specific adsorption of target molecules, one should exclude undesired changes caused by the bulk fluid, which would induce changes in refractive index around the sensor. For this reason, it is necessary to know the relationship between critical angle and resonance angle. The present study monitored the changes in the critical angle and resonance angle on full SPR curve using a DIW and a glycerol-water solution with a concentration in the range of 1% to 5% as a refractive-index solution. In a Figure 7a, the black dotted lines are SPR full curves measured for samples with each concentration of glycerol-water solution and the red solid lines represent fitting curves obtained by sigmoid-asymmetric method. A critical angle and a resonance angle on each curve were determined by the presented algorithm. Figure 7b presents a correlation between the critical angle and resonance angle caused by the change in the fluid refractive index due to the glycerol-water solutions. Fortunately, the correlation represents a simple linear equation in the range of 0.5613°, which is sufficient to measure biomolecular interactions among two or three macromolecular layers in real time as discussed in a previous work [26]. The slope of the trend line in the plots was 0.97, and the coefficient of determination was 0.999. Thus, we determined the normalization constant as 0.97, and the final equation for the specific adsorption angle in our system is described as follows:

(5) $$\theta_{SAA}=\;\theta_{RA}-\;0.97\theta_{CA}$$

where *θ_SAA_*, *θ_RA_*, and *θ_CA_* indicate the specific adsorption angle, resonance angle, and critical angle, respectively.

**Figure 8:**
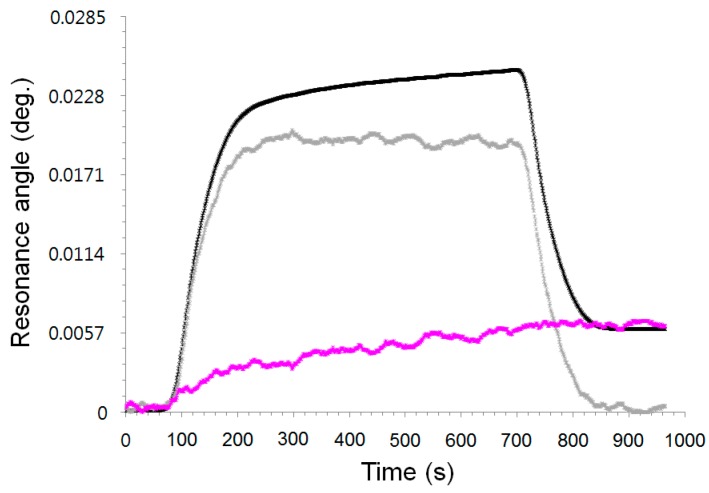
Sensorgrams obtained by measuring *θ_RA_* (black solid line), *θ_CA_* (gray solid line), and *θ_SAA_* (pink solid line) in real time on binding, diluted BSA-glycerin mixture in PBS buffer solution on bare gold sensor chip.

We conducted additional experiments for protein adsorption in real time to confirm the feasibility of removing the bulk fluid effect using the novel sigmoid-asymmetric equation-based algorithm. The black solid line of Figure 8 represents a sensorgram for measuring the corresponding change in the resonance angle on the BSA adsorption as a measurement method of the conventional SPR system. First, the PBS buffer solution was injected into a micro channel on the gold sensor chip for a baseline. Then, the BSA solution was loaded at the 80 s point. The change in the resonance angle dramatically increased until 200 s. This change reflects a mass increase by the adsorption of the BSA on the surface and the change in the bulky refractive index due to the glycerin concentration. The change in the resonance angle then increased sluggishly until the 700 s point. This change includes only the mass change due to the adsorption of the BSA on the gold surface via hydrophobic interaction. Finally, the change in the resonance angle was dramatically reduced from the 700 s by washing with a PBS buffer solution. The signal was stable at a higher position than the baseline: ~0.0285°. This value indicates the specific adsorption level of the BSA on the gold sensor chip. If we do not know the composition of the sample solution, we cannot understand the meaning of step-by-step changes in the resonance angle.

The sensorgram can be interpreted in many ways. For example, we can attribute the increased signal to the binding of abundant BSA, including strong and weak binding on the gold sensor surface. We can also predict a dramatic decrease in the signal due to the desorption of the weak-binding BSA on the surface. The gray solid line of Figure 8 represents a sensorgram for measuring the change in the critical angle.

We observed that the baseline before loading the BSA-glycerin solution was the same as the last position after the washing with the PBS buffer solution. This sensorgram indicates only the change in the bulky refractive index around the sensor. Finally, we observed a sensorgram to evaluate only the change of specific adsorption angle, *i.e.*, the change in the critical angle caused by the bulky refractive index subtracted from the change in the resonance angle obtained using Equation (5), indicated by the pink solid line in Figure 8. Its value is slowly increased by the adsorption of the BSA before the washing with the PBS buffer, and it is maintained after the washing, not decreasing due to the desorption. We confirmed that the sensorgram is very different from it regarding the change in the resonance angle due to the elimination of the change caused by the bulk refractive index.

We successfully implemented a sensorgram for measuring the specific adsorption angle by conducting a protein adsorption experiment using only the novel fitting method with a self-constructed wedge-shaped beam type angular interrogation SPR spectroscopy, without any referencing approach or time consuming multi-layer Fresnel equation. We also consider that the sigmoid-asymmetric equation based full SPR curve fitting method is practically useful for the simultaneous and automatic determination of the critical angle and resonance angle in real time. 

## 4. Conclusions

In this study, we introduced a novel full-SPR-curve-fitting algorithm based on a sigmoid-asymmetric equation that can rapidly determine the critical angle and resonance angle in real time. The fitting curves obtained by the proposed sigmoid-asymmetric based approach almost perfectly matched the full SPR curves with water and air as bulk fluids on the sensing film. This was also proven with the available fit quality parameters, which were better than those obtained using fitting methods that are conventionally used to determine the optimal resonance angle, including the error variance and coefficient of determination. The novel algorithm effectively eliminated the undesired change caused by the bulk fluid refractive index change on the sensorgram for measuring the molecular interaction. As a result, we realized a sensorgram for measuring the specific adsorption angle without changes caused by the bulk refractive index, by subtracting the critical angle from the resonance angle in real time using a sigmoid-asymmetric fitting algorithm. We consider that the sigmoid-asymmetric-equation-based full-SPR-curve-fitting method is practically useful for the simultaneous and automatic determination of the critical angle and resonance angle in real time in various applications including gas sensing and solutions based sensing. We believe that the sigmoid-asymmetric fitting equation can be applicable to commercially available SPR systems.
